# Plan to Insure Actual Attendance upon Medical Lectures

**Published:** 1848-03

**Authors:** 


### EDITORIAL DEPARTMENT.

*Plan to insure actual attendance upon Medical Lectures.*—Among the resolutions appended to the report of the Committee on requirements for a degree, made at the second National Convention, and adopted by that body, is the following:—"That it be suggested to the Faculties of the various Medical Institutions of the country, to adopt some efficient means for ascertaining that their students are actually in attendance upon their lectures."

We are not aware of any Institution having taken action upon the subject of the above resolution, nor have we met with any remarks upon it in any of the Journals which we receive. The subject must, however, have appeared, both to the Committee, and the Convention, to possess some importance, else the resolution would not have been reported by the one, nor been adopted by the other. So far as our observations and knowledge of Medical Institutions extend, students in attendance at Lectures are generally anxious to avail themselves of the advantages thereby afforded, and are faithful in attendance, and attentive, during the whole course. But there will be some members of every class who are neglectful in both particulars. To some extent, also, the custom prevails of shortening attendance by not being promptly present at the commencement of the session, and by leaving before the courses are completed. The resolution referred to, doubtless, was aimed at these evils. That they are evils of no small magnitude must be conceded on all hands. The only point for discussion is, how are they to be remedied. The resolution recommends to the Faculties of Medical Schools to devise and carry into effect some efficient means of prevention; and if any means are at their disposal, they are bound to employ them for the desired end. It is, of course, practicable to ascertain if students are in daily, regular attendance upon the different lectures. This may be done by *calling the roll* or by a monitorial system, such as is pursued in Academic Institutions. But the purpose is obviously not answered by merely ascer taining whether students are in attendance, or not. The attendance must be rendered obligatory, and in some way secured. The following plan has suggested itself to us as adequate to the purpose, and, if adopted by Institutions in concert, no objections can arise to its being carried into effect:—The plan is simply to give at the close of the term certificates of attendance upon the several branches, respectively, to those who have actually and faithfully attended, these certificates being essential, as evidence of having attended lectures, before pupils are examined for degrees. The plan proposes to substitute for the tickets now issued at the begining of the term, and which are now recognized as sufficient evidence of having attended the prescribed courses of lectures, certificates granted, when fairly earned, at the close of the term. Tickets admitting students to the lectures may, of course, be given as they now are, and should be paid for when received, but they should not be taken as evidence that the bearers have actually attended the lectures. They cannot really, in any case, constitute such evidence, for they are issued before the lectures commence. We venture to suggest this method of carrying into effect the recommendation of the Convention, and we can perceive no difficulty in the execution, provided schools, generally, will unite in adopting and adhering to it.

